# Supplementary material for: StayGold photostability under different illumination modes
Source: Sci Rep. 2024 Mar 6;14:5541. doi: 10.1038/s41598-024-55213-3 (PMC10918099; doi:10.1038/s41598-024-55213-3)
Supplement: Supplementary file 1 — Supplementary Figures. [file 41598_2024_55213_MOESM1_ESM.pdf]

Supplementary Information

StayGold Photostability under Different Illumination Modes

Masahiko Hirano, Yasuo Yonemaru, Satoshi Shimozono, Mayu Sugiyama, Ryoko Ando, Yasushi Okada, Takahiro Fujiwara, Atsushi Miyawaki

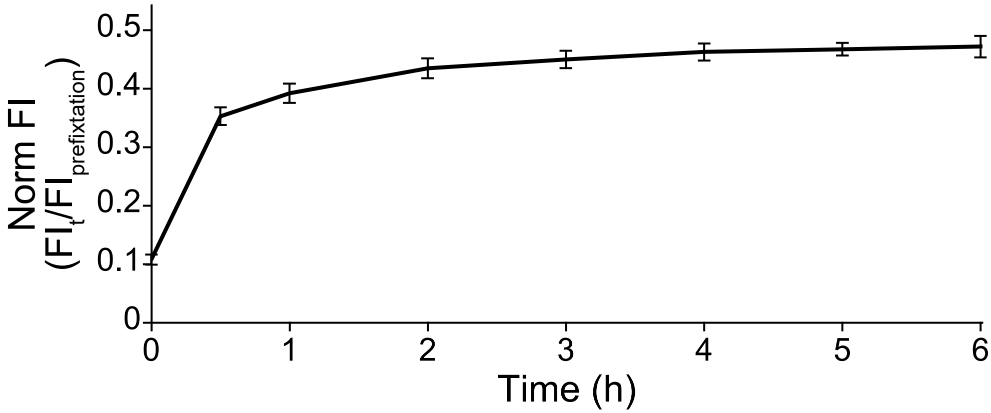

Supplementary Fig. 1 | Recovery of mNeonGreen fluorescence in fixed HeLa cells after washing with HBSS.

Data points are shown as means ± SD (n = 5 cells). See Figure 2 (H2B-mNeonGreen).

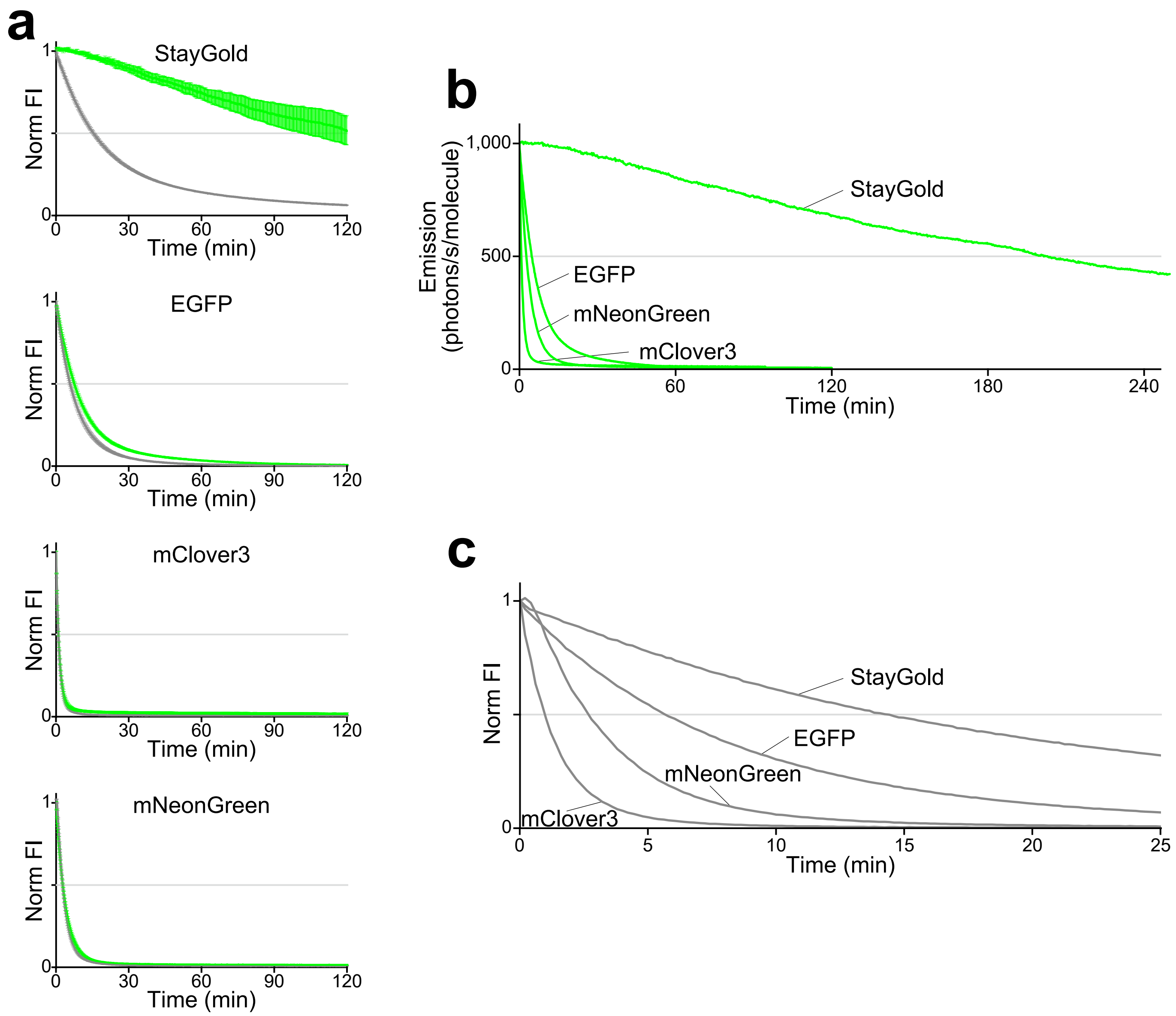

Supplementary Fig. 2 | Photostability of StayGold, EGFP, mClover3 and mNeonGreen fused to H2B in living and fixed HeLa cells under continuous WF illumination.

Irradiance: 1.9 W/cm^2 for EGFP, mClover3 and mNeonGreen; 1.8 W/cm^2 for StayGold.

(**a**) Photostability of the four green-emitting FPs in living (green lines) and fixed (gray lines) cell samples. Photobleaching curves were simply normalized; in each experiment, $FI_{(t)}/FI_{(0)}$ was plotted against time.

(**b**) Photostability in living cells. Photobleaching curves are calculated based on the FP molecular brightness and irradiance (Table 1), plotted as intensity versus normalized total exposure time with an initial emission rate of 1,000 photons/s/molecule. The results were similar to those obtained in our previous WF illumination experiments that monitored the fluorescence of StayGold, EGFP, mClover3 or mNeonGreen distributed throughout the cytosolic and nuclear compartments (ref. 5).

(**c**) Photostability in fixed cells with an irradiance value of 1.9 W/cm^2. The molecular brightness of FPs in their fixed state was not determined. Accordingly, photobleaching curves were based only on irradiance. In each experiment, $FI_{(t)}/FI_{(0)}$ was plotted against time. The coordinate time for StayGold was scaled by 0.947 (=1.8/1.9).

(**a** and **c**) Cells were treated with 4% PFA for 30 min.

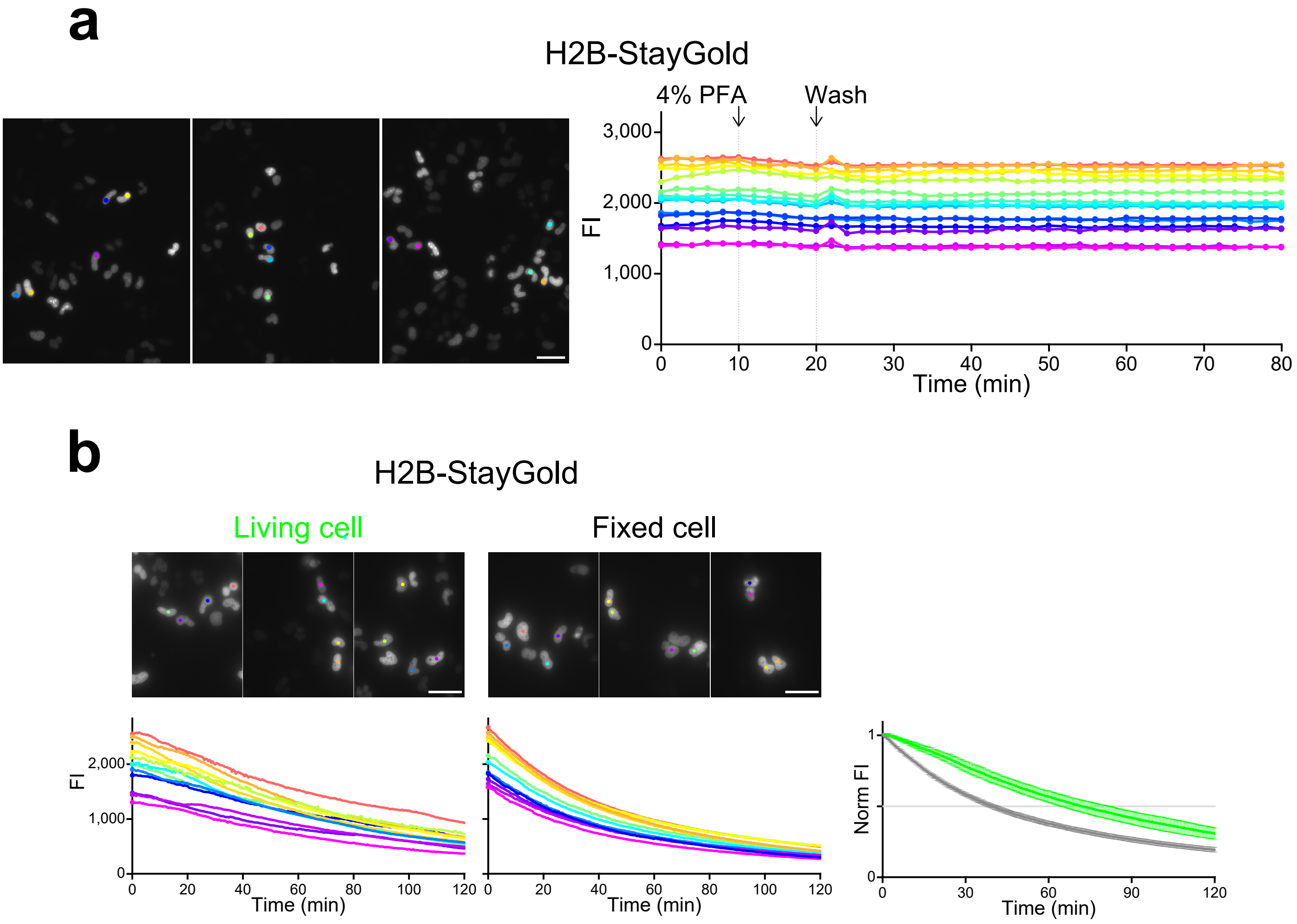

Supplementary Fig. 3 | Photostability of nuclear-targeted StayGold before and after 10-min fixation.

(**a**) Time-lapse imaging of HeLa cells expressing H2B-StayGold. *left,* Representative low-magnification fluorescence images before fixation. Scale bar, 50 µm. *right,* FIs of several cells (indicated in the first images, *left*) are individually plotted against time. Treatment with 4% PFA was conducted for 10 min.

(**b**) *left,* Photobleaching curves of StayGold in live-cell samples. *middle,* Photobleaching curves of StayGold in fixed-cell samples. In each photobleaching experiment, tweleve cells were observed (indicated in the first images, *top,* scale bars, 50 µm). *right,* Photobleaching curves (green lines, live cells; gray lines, fixed cells) were simply normalized; $FI_{(t)}/FI_{(0)}$ was plotted against time. Data points are shown as means ± SD (n = 12 cells).
